# Diagnosis of Irritable Bowel Syndrome: Role of Potential Biomarkers

**DOI:** 10.1155/2015/490183

**Published:** 2015-06-11

**Authors:** Ivana Plavšić, Goran Hauser, Mladenka Tkalčić, Sanda Pletikosić, Nermin Salkić

**Affiliations:** ^1^Emergency Department, Clinical Hospital Centre Rijeka, Krešimirova 42, 51000 Rijeka, Croatia; ^2^Department of Gastroenterology, Clinical Hospital Centre Rijeka, Krešimirova 42, 51000 Rijeka, Croatia; ^3^Department of Psychology, Faculty of Humanities and Social Sciences, University of Rijeka, Sveučilišna Avenija 4, 510000 Rijeka, Croatia; ^4^Department of Gastroenterology and Hepatology, University Clinical Centre Tuzla, Trnovac bb, 76000 Tuzla, Bosnia and Herzegovina

## Abstract

Irritable bowel syndrome is a disorder diagnosed on symptom-based criteria without inclusion of any objective parameter measurable by known diagnostic methods. Heterogeneity of the disorder and overlapping with more serious organic diseases increase uncertainty for the physician's work and increase the cost of confirming the diagnosis. This paper is an attempt to summarize the efforts to find adequate biomarkers for irritable bowel syndrome, which should shorten the time to diagnosis and reduce the cost. Most of the reviewed papers were observational studies from secondary care institutions. Since publication of the Rome III criteria in 2006, most recent studies use these for the recruitment of IBS patients. This is a positive step forward as future studies should use the same criteria, facilitating comparison of their results. Among the studied biomarkers, most evidence is provided for fecal calprotectin. Cutoff values for fecal calprotectin have still to be investigated prior to inclusion in the irritable bowel syndrome diagnostic algorithm.

## 1. Introduction

Irritable bowel syndrome (IBS) is defined as a functional disorder of the lower gastrointestinal (GI) tract, which manifests as abdominal pain and/or discomfort accompanied by altered bowel function, in the absence of biochemical or structural pathology [1]. According to the NICE guidelines, prevalence of IBS is between 10 and 20% worldwide, with twice the prevalence in women compared with men [2]. The need for a reliable and standard method to properly discriminate functional gastrointestinal disorders (FGIDs) has led to the development of symptom-based criteria by the Rome Foundation. Accordingly, diagnosis of IBS is established on clinical background with exclusion of “red flag” symptoms (age > 50, rectal bleeding, anemia, short-term symptoms, and weight loss). Patients with IBS are divided into subgroups based on their predominant symptoms: diarrhea predominant (IBS-D), constipation predominant (IBS-C), mixed type with diarrhea and constipation (IBS-M), and undetermined IBS. Around 75% of patients are alternators, which illustrates the instability of symptoms over time in the same patient [3]. It is confirmed that IBS is not associated with the development of serious disease or increased mortality but nonetheless every patient with IBS is routinely checked for more serious diseases such as inflammatory bowel disease or gastrointestinal carcinoma. Patients are exposed to a set of invasive tests, despite the opinion that less than 5% of patients with a diagnosis of IBS based on the Rome III criteria are likely to have symptoms caused by a more serious condition [4]. The reason for the excessive use of invasive diagnostics lies in the complex pathophysiology of the disorder, resulting in symptoms that often overlap with organic diseases that require early recognition and treatment. IBS carries a great psychological burden for patients, who usually consume more medications, miss more workdays, have low work productivity, and are hospitalized more frequently. Consequently, IBS becomes not only debilitating for the patient, but also a great social and economic burden [5]. Noninvasive markers for IBS have paramount importance because unresolved pathophysiology and conflicting results present an insurmountable obstacle in reducing the cost of diagnosis and treatment of patients with IBS. Our primary aim was to review the most important ideas and findings regarding the pathogenesis of IBS and the second aim was to hypothesize biomarkers that may one day present the “gold standard” in diagnosing this complex disorder.

After completing an extensive review of the literature on pathophysiology and diagnosis of IBS we now present findings along with conclusions.

## 2. Pathogenesis of IBS

### 2.1. Environmental Contribution and Genetics

Many researches list various reasons for IBS development. One of the hypothesized reasons is the role of genetics and environmental contribution. Saito et al. reviewed the literature on the subject and concluded that published data suggest evidence of genetic susceptibility to IBS. Their conclusion is based on familiar aggregation studies, twin studies, and pharmacogenomic studies. They also mention polymorphism of the serotonin transporter gene in IBS, which to date is probably the most intriguing finding due to the direct link between serotonin and gut motility and mood [6]. Zhang et al. confirmed that the LL genotype of the serotonin transporter gene is involved in higher risk of IBS-C development with no effect on other subtypes. They claim that their finding is applicable to the East Asian population and not to other populations [7]. In addition, more than 60 genes have been linked to IBS. Most of these are associated with inflammation, neurotransmitters, and bile acid synthesis [8]. Although findings in gene research are incoherent and sometimes contradictory, further research with larger samples should be conducted because of the undisputed role of gene expression in disease phenotype, identification of the objective marker, and appropriate therapy.

### 2.2. Low-Grade Inflammation

For many years, IBS was considered to be an exclusively functional disorder. Therefore, the diagnosis is based on symptoms and exclusion criteria for more serious conditions. Recent studies have focused more on a low-grade inflammation found in a subset of patients with IBS. Chang et al. consider results of low-grade inflammation to be more consistent in patients with postinfectious IBS (PI-IBS) than in those with non-PI-IBS [9]. These findings are interesting because of the possibility of developing a specific biomarker for IBS that will help clinicians to distinguish IBS from other FGIDs and more serious organic diseases with great sensitivity and specificity. In 2009, Cremon et al. confirmed low-grade inflammation in the lamina propria and mucosa in 72% of patients with IBS but to a lesser extent than in microscopic colitis or ulcerative colitis. Although they tried to connect types of inflammatory cells to certain symptoms in IBS, correlation could not be found. A possible reason is that several IBS symptoms can be correlated just with a too high somatization rate or some other psychosocial factors, but not to inflammatory processes [10]. The authors managed to show a statistically relevant connection between mucosal mast cell infiltration and frequency of abdominal bloating [11]. This finding can be due to anatomical and functional communication between mast cells and intrinsic and extrinsic nerves in charge of sensorimotor function of GIT, rather than to an increased number of mast cells alone [12]. Mast cell infiltration is found throughout the GI tract and its role in inflammation and symptom development has attracted most attention [13]. Barbara et al. confirmed that acute stress induces mast cell infiltration and activation in the gut but the role of chronic stress is not yet fully investigated [12]. Besides mast cells in the mucosa of IBS patients, other kinds of inflammatory cells can be found. Among them are T-lymphocytes which are representative of adaptive immunity and have a role in activation of B-lymphocytes and macrophages [14]. Another finding that supports immune activation is the increased number of proinflammatory cytokines in the serum of IBS patients. Dinan et al. showed an increased number of IL-6 and IL-8 in IBS subgroups (diarrhea predominant, constipated, and alternators) [15]. Cytokine imbalance and presence of various inflammatory cells are proven, but the question that remains is the missing link that directly connects prementioned findings and IBS symptoms. This is why an increased number of mast cells and their supposed role in certain IBS symptoms are the most consistent finding so far [11, 16].

### 2.3. Postinfectious Low-Grade Inflammation

The term “postinfectious IBS” was used as a term for the first time in a study in the 1950s [17]. It is considered that around 10% of patients with infectious gastroenteritis (GE) experience persistent symptoms that coincide with IBS diagnosis criteria [18]. The exact reason why some patients fully recover and why infectious GE in others progresses to PI-IBS is not fully understood. A supposed underlying mechanism is genetic polymorphism in genes associated with immune response to pathogens and immune functioning of the individual that allows activation of a specific inflammatory response [19, 20]. Low-grade inflammation through the presence of different immune cells such as leukocytes, lymphocytes, and mast cells in this subtype of IBS patients is mentioned in different studies [21, 22]. Thus, low-grade inflammation is considered to be the main pathophysiology of PI-IBS. It would be interesting to set up a study to investigate how long the symptoms of IBS persist in this subgroup of patients and to find a correlation between the grade of infective acute GE and subsequent symptoms of IBS. But there are many limitations to this type of research because it is mainly retrospective and highly dependent on patients' recall of symptoms of past GE infection [23].

### 2.4. Microbiota

The role of intestinal microbiota in IBS pathogenesis is another interesting field of research. Some authors, in referring to intestinal microbiota, use the term “virtual organ,” highlighting its importance. Altered fecal microbiota is described in a subset of patients with IBS compared with healthy controls [24, 25]. Kennedy et al. mention direct and indirect evidence of altered microbiota. Direct evidence is based on the search for different types of bacteria in the gut and feces and monitoring changes in their number. The results showed an increase in the number of certain subtypes at the expense of other types of bacteria in patients with IBS compared with healthy controls. The finding imposed an opinion of how reduced diversity of microbiota in the gut is shown in IBS patients. Since it is considered that stable but diverse microbiota is beneficial to health, these changes in the composition of microbiota can result in IBS symptoms [26]. A direct link has not been found although many researches are trying to investigate the incidence of symptoms and special species of bacteria [27]. Indirect evidence of the role of microbiota is based on the observed beneficial effect of probiotics on IBS symptoms [28]. On the other hand, increased usage of broad spectrum antibiotics increases the prevalence of IBS [29]. Another assumed link between microbiota and IBS is the existence of PI-IBS [30].

### 2.5. Neuroendocrine System (Brain-Gut Axis) and Endocrine-Immune Axis

The brain-gut axis (BGA) consists of the peripheral neuroendocrine system, central nervous system, particularly the autonomic nervous system, and the hypothalamic-pituitary-adrenal (HPA) axis [31]. The BGA controls motility, secretion, absorption, microcirculation, local immune defense, and cell proliferation. Fichna and Storr mentioned that bidirectional communication along the neural, endocrine, and neuroimmune pathways allows signals from the GI tract to influence the brain, which in turn can exert changes in motility, secretion, and immune function [32]. Dysregulated BGA is a theoretical construct, which serves as an explanation for the changes in visceral hypersensitivity and gut motility, as the two main pathophysiological causes of IBS symptoms [33]. Motor and sensory functions are controlled by the enteric endocrine system which releases signaling molecules. Two major products of the enteric endocrine system are serotonin (5-HT) and the chromogranins (Cgs) family [34]. Enterochromaffin cells (EC) are dispersed throughout the GI mucosa and present the main source of biogenic amine 5-HT in the gut. Around 95% of the body's 5-HT is produced in the GI tract [35]. 5-HT is released in a regulated manner in response to various mechanical and chemical stimuli, including bacterial stimuli [35]. Several studies have reported changes in the EC population and 5-HT content in inflammatory bowel disease (IBD) and in IBS [36]. Coates et al. showed that 50% of patients with IBD in long-standing remission have IBS-like symptoms [37]. The basis for this conclusion is low-grade inflammation that alters the normal 5-HT signaling cascade producing chronic IBS-like symptoms [38]. 5-HT has the ability to regulate inflammation by acting on signaling pathways, by inducing production of inflammatory mediators from immune cells and mediating interaction between the innate and adaptive immune response. Its role in IBS pathogenesis is one of the most investigated so far.

The family of granins is ubiquitous in the enteric, endocrine, and immune systems. It regulates a number of cellular functions, including packing of hormones and formation of secretory granules. Chromogranins and secretogranins as representatives of the granins family serve as precursors for several bioactive peptides with different bioactive functions [39]. At present, CgA is used as a marker for neuroendocrine tumors since it represents overall activity of the endocrine system in the body [40]. Recently, scientists have focused a great deal of attention on the chromogranins family since it was revealed that it also has a role in the pathogenesis of IBS and may serve as a potential biomarker for the disorder.

## 3. Inflammatory and Endocrine Biomarkers for IBS

To reduce costs and to minimize unnecessary diagnostic tests, there is a growing need for the discovery of a valid biomarker. In 2001, the term “biomarker” was defined as “a characteristic that is measured and evaluated as an indicator of normal biological processes, pathogenetic processes or pharmacologic responses to a therapeutic agent” [41]. Connor and Leonard describe the ideal biomarker as “one that exhibits accuracy, reproducibility, sensitivity, specificity and patient acceptability” [42]. The greatest obstacles in finding biological biomarkers in IBS are heterogeneity of symptoms between patients and temporal instability of the symptoms in the same patient, also overlapping of symptoms with other functional gastrointestinal disorders (FGIDs) and more serious organic diseases, and, finally, unclear understanding of the pathophysiology of disorders [43]. Regarding the aforementioned problems, it is unrealistic to expect a single biomarker candidate to be applicable to all aspects of the disorder [43]. As Bennike et al. consider in their study, intestinal tissue is an obvious place to search for novel biomarkers in IBD and the same logic should be valid for IBS also. Upon confirmation of the biomarker, samples such as blood, urine, and stool should in future be screened for this [44]. The search for biomarkers in IBS is predominantly based on the assumed pathogenetic mechanisms described in the text above. Serum biomarkers are represented in the form of C-reactive protein (CRP), erythrocyte sedimentation rate (ESR), cortisol levels, and serum levels of chromogranin and different proinflammatory cytokines. They are all highly unspecific for IBS and can be viewed as general markers of inflammation or general endocrine activity in the case of chromogranin levels. CRP is one of several proteins in which production in the liver is intensified in the acute phase of inflammation due to stimulation of proinflammatory cytokines. CRP is usually used to assess the degree of inflammation and therapeutic success in diseases such as Crohn's disease and ulcerative colitis, once the diagnosis is made. Hod et al. tried to confirm the hypothesis of elevated high sensitivity CRP (hs-CRP) as a marker of microinflammation in IBS. Values of hs-CRP in patients with IBS were in the normal laboratory range but still a significant difference in hs-CRP values between patients with IBS and healthy volunteers was noticed. They concluded that such a finding can support the existence of microinflammation [45]. The clinical relevance of CRP values in assessing IBS disease severity or therapy follow-up has not yet been proven.

As with CRP, erythrocyte sedimentation rate (ESR) is also hypothesized to be a nonspecific marker for microinflammation [46]. Authors see an advantage in using ESR due to its simplicity and improvement of initial therapy approach. It would be easier to choose treatment for patients if those with predominance of mucosal inflammation were distinguished from those with more psychosocial disturbances [46]. Cortisol is a glucocorticoid, produced by the zona fasciculata of the adrenal cortex. The level of cortisol in the blood depends on HPA axis activity. Although stress is not the only reason for higher cortisol release into the bloodstream, it is widely known as “the stress hormone.” Early life trauma and chronic stress are confirmed as major risk factors for IBS; therefore the idea of measuring cortisol levels in these patients and searching for disturbances in the HPA axis seems a logical way of proceeding with research into the origin of disorders [47, 48]. Recently, Kennedy et al. performed a study in which they, among others, measured salivary cortisol levels in response to the Trier Social Stress Test (TSST). They found greater total cortisol output in response to acute stress in IBS patients and concluded that the cause lies in failure of the HPA axis to adequately shut off long after the stressor has been removed and generalized elevation in HPA axis activity [49]. A similar study was performed in 2009, in which authors measured cortisol levels in women with IBS-D after lumbar puncture as representative of a physical stressor. Results of this study showed an attenuated response of the HPA axis in patients with IBS compared with healthy controls. The impaired tone of the HPA axis was attributed to adaptive changes in brain response to chronic stress to which IBS patients are considered to be more often exposed in comparison with healthy individuals [50]. Although evidence for altered HPA axis in response to different stressors among IBS patients exists, measurement results of basal cortisol levels are still conflicting and incoherent. There are many different variables that need to be taken into account in such research, such as gender, age, presence of psychiatric comorbidity, menstrual cycle in female patients, type of stressor, type of IBS, and environmental and genetic influences.

Analyzing more than 600 different pathways and 60 000 different biomarkers that connect a single gene to physiological processes in GIT and could be linked to development of IBS and other GI disorders, Lembo et al. selected potential blood-based biomarkers to differentiate IBS from non-IBS functional and organic disease. Final biomarkers included interleukin-1*β*, macrophage inflammatory protein-3*β*, growth-regulated oncogene *α*, tumor necrosis factor-related weak inducer of apoptosis, neutrophil gelatinase-associated lipocalin, osteoprotegerin, vascular endothelial growth factor, brain-derived neurotrophic factor, metalloproteinase-9, and tissue inhibitor of metalloproteinase-1. They are included in a diagnostic test shown to have 50% sensitivity and 88% specificity in differentiating IBS from non-IBS patients [51].

The chromogranins family recently became popular in the search for the ideal biomarker for IBS since it was discovered that they can modulate intestinal inflammation and present active communication between the neuroendocrine and immune system [52]. Sidhu et al. found an elevated chromogranin A (CgA) serum level in a subset of IBS-D patients [53]. The authors suggest that this finding could be the result of enterochromaffin cell hyperplasia found in PI-IBS patients [54, 55]. Since inflammation is one of the main underlying causes of PI-IBS it has been considered that inflammation could induce EC hyperplasia and result in an elevated chromogranin level. The role of chromogranin as an inflammation marker has yet to be proven. In their study, only prospectively selected IBS-D patients were included and no other group of IBS patients was involved. Also, a significantly elevated level of chromogranin was transient in a small number of patients. Therefore, studies on a larger number of patients need to be conducted to find the clinical relevance of an elevated CgA serum level. In contrast, El-Salhy et al. found no increased CgA blood level compared with healthy controls and considered that changed levels of chromogranin A in blood are clinically insignificant. Instead, they found reduced density of chromogranin A-containing cells in the duodenum and colon of both IBS-D and IBS-C patients [56]. Due to this finding, they propose changed density of intestinal CgA cells as a potential histopathological marker for IBS [56]. Until the results of a study on a larger population of patients are published, we can only rely on the knowledge that serum levels of CgA present a reliable marker for neuroendocrine tumors. Perhaps negative values of serum CgA could be included in the IBS diagnostic algorithm to eliminate the possibility of endocrine tumors in the gastrointestinal tract. Since stool is in direct contact with the intestinal wall, it can be analyzed in the search for GIT inflammation. Öhman et al. searched for altered chromogranins and secretogranins levels in stool samples of IBS patients. This involved predominantly patients with IBS-D and IBS alternators (IBS-A) and the authors found elevated levels of CgA and secretogranins II and III [39]. The most important observation is a strong negative correlation between the colonic transit time and fecal levels of mentioned granins. Such a discovery opens the door to new questions and hypotheses regarding the role of fecal granins in IBS. Some are discussed by Camilleri who wonders whether these elevated levels are a cause or just a consequence of GIT disturbances in IBS [57]. Fecal and serum granins can be elevated in various conditions, so lack of specificity and clear distinction between their levels in IBS and healthy volunteers do not support them as positive biomarkers for IBS so far.

One of the most studied biomarkers in stool samples is fecal calprotectin (FC). It is a calcium-binding heterodimer and can be found in the cytoplasm of neutrophils. It is released into the intestine during colonic inflammation and is resistant to colonic degradation so it can easily be measured in stool [58]. The relevance of FC is in the correlation of its level in stool sample and the degree of GIT inflammation. In addition, it correlates with endoscopic criteria for inflammation severity. The clinical utility of such a finding lies in easier and cheaper monitoring of the disease severity index and therapy effectiveness in patients with proven IBD [59]. Newer studies are trying to find relevant cutoff FC stool levels that can with great certainty distinguish IBS from IBD and reduce unnecessary invasive diagnostic tools. The foundation for such research lies in the discovery that IBS symptoms persist in a large number of patients with CD and UC who are considered to be in remission [38]. The FC level in these groups of patients is found to be much higher than in IBS patients. That means that we can use FC as a valid biomarker for the discovery of low-grade inflammation in IBD [60]. Most researches do not consider that neutrophil infiltration is a feature of the immunoinflammatory changes in the mucosa of patients with IBS and found FC levels in IBS to be within normal range [61]. Trying to define IBD exclusion criteria, Tibble et al. mention that cutoff FC levels of 30 mg/kg combined with Rome I criteria can serve as a clear proof of IBS with no need for further examination [61]. Similarly, in a report published in 2013, fecal calprotectin is confirmed as a highly specific and sensitive biomarker for IBD and a value from 50 mcg/g showed 93% sensitivity and 94% specificity in differentiating IBD from IBS [62]. As with CRP values, low values of FC can serve as proof of microinflammation and confirmation of the pathological process developing in the GI tract of examined subjects. Chang et al., who found significantly higher stool FC levels in IBS patients than serum CRP levels, presented an interesting finding [63]. Findings of elevated FC should be investigated further,because it may increase the sensitivity and specificity of tests performed in the diagnostic algorithm to confirm IBS. Another positive remark on FC is the opinion that FC level correlates with a reduced physical component of health related quality of life (HRQoL) [64]. This means that it can be used to monitor the response to therapy, as in IBD. Measurement of FC level in stool should be included in the IBS diagnostic algorithm, regardless of whether it is used to confirm microinflammation and to choose an adequate therapy approach for these patients or to exclude the diagnosis of IBD and minimize unnecessary invasive procedures.

Another fecal marker indicative for inflammation is human *β*-defensin-2 (HBD-2). This peptide is produced by colonic tissue and is considered part of the innate immune system. It scarcely exists in uninflamed colon. Production is dependent on the microorganism's activity and proinflammatory cytokines. Langhorst et al. found significantly higher levels of HBD-2 in patients with IBS compared with healthy controls. They consider such a finding supportive of the proinflammatory response in IBS patients in the absence of macroscopic signs of inflammation [65]. HBD-2 presents another potential biomarker whose clinical role in IBS has not been adequately investigated so far. In the future, the search for biomarkers should also be based on ideas that arise from the development of new technologies. A promising approach in biomarker discovery is the examination of the proteome (proteomics). Proteomics is the determination and quantification of the complete protein content in the human body at any specific moment. It focuses on cellular and secreted proteins, in terms of both their structure and the functional interaction between them [44]. In one study, the authors used animal models for IBS and examined colonic tissue proteomics. Out of 1396 proteins, 13 were differently expressed and they were all connected to intestinal tract immunity and nerve regulation [66]. A further step should be identification of targeted proteins and observation of their expression pattern when a noninvasive sample is used (blood, feces, and urine) [67].

## 4. Conclusion

The definition of IBS is so far a theoretical construct, diagnosed and treated in a similar way to psychiatric disorders. Because of the low risk of developing serious GIT disease and high costs for the healthcare system worldwide, IBS should be diagnosed and treated by primary care physicians. Minimizing the cost of diagnosing IBS implies identifying patients with low risk of organic disease by combining symptom-based criteria and inexpensive laboratory tests available to primary care physicians. Heterogeneity of symptoms between patients and even within the same patients over time and overlapping symptoms with serious organic diseases and other functional gastrointestinal disorders, combined with unclear understanding of the pathophysiology of the disease, present a problem for researchers and their attempt to establish a universal test to confirm the diagnosis of IBS. According to Jones et al., development of a reliable biomarker panel is complicated by the absence of a perfect reference standard [68]. The finding of low-grade inflammation in IBS has led to research in inflammatory biomarkers as a possible end-point for the establishment of diagnosis and drug development. Confirmation of low-grade inflammation in a subgroup of patients can help physicians to discriminate between patients with predominance of mucosal inflammation and those with more psychosocial disturbance and assist in the selection of appropriate therapy. Most immunologic and endocrine biomarkers are highly unspecific. As shown in Table 1, most of the studies reviewed for the purpose of this paper are solely observational which reduces the quality of the evidence. Measurement of fecal calprotectin in stool samples was shown to be most promising in differentiating between inflammatory bowel disease and IBS. Adequate cutoff levels, which could firmly exclude IBD and also confirm microinflammation in IBS, are not widely accepted.

## Figures and Tables

**Table 1:** Biomarkers for IBS diagnosis.

Authors	Study design	Methods	Population	Number of IBS patients	Biomarker	Main results
Hod et al. (2011) [45]	Case control	Prospectively selected IBS patients diagnosed according to *Rome III criteria.* All patients underwent blood sampling for hs-CRP levels	IBS (IBS-C, IBS-D, IBS-M) compared to healthy controls	88	hs-CRP levels are higher in IBS patients than HC but still in the normal laboratory range	Mean hs-CRP level in patients with IBS versus healthy controls (1.17 ± 1.26 mg *L* (−1) versus 0.72 ± 0.91 mg *L* (−1), resp. (*p* = 0.001))

Hauser et al. (2012) [46]	Pilot study	IBS was diagnosed based on *Rome III criteria.* Patients underwent a medical and laboratory examination (ESR), FBDSI^1^ and filled in the questionnaires designed to measure their disease-specific and general HRQoL: (IBS-36)^2^ and (SF-36)^3^	IBS (all patients were included despite disease's subtype or previous enteric infectious status)	86	IBS patients with higher ESR (>24 mm/h) expressed lower disease-specific quality of life	ESR positively correlated with disease-related HRQoL (*r*_85_ = 0.24, *p* < 0.05)

Kennedy et al. (2014) [49]	Observational study	Female IBS participants who met *Rome III criteria* with exclusion of organic GI disease underwent a single exposure to the TSST^4^. Salivary cortisol, salivary CRP, SCL^5^, GI symptoms, mood, and self-reported stress were measured	IBS (female) compared to age-related female healthy controls	13	Patients with IBS exhibited a greater total *cortisol* output and exhibit sustained HPA axis activity to acute experimental psychosocial stress	Repeated measures ANOVA across all sample collection time points revealed a significant main effect of the TSST on salivary cortisol levels (*F*_4.16,108.11_ = 8.34, *p* < 0.001, *ηp* ^2^ = 0.243) and a significant main effect of group (*F*_1,26_ = 4.96, *p* = 0.035, *ηp* ^2^ = 0.16)

FitzGerald et al. (2009) [50]	1-day observational study	All patients with IBS fulfilled *Rome II criteria* IBS-D and none of the patients were classified as PI-IBS. Blood samples were analyzed for ACTH^6^, cortisol, epinephrine, and norepinephrine. A single measure of cerebrospinal fluid (CSF) concentrations of corticotropin-releasing factor (CRF_CSF_) and norepinephrine CSF is noted	IBS-D (women) compared to healthy controls	13	No CRF_CSF_ differences between groups are observed. Women with IBS display blunted ACTH and cortisol responses to the acute stressor (lumbar puncture)	The mean CRF_CSF_ was 23.9 (SE = 6.14) in patients with IBS compared with 23.0 ± (SE = 2.23) in controls. A significant interaction of time × group was seen for ACTH, *F* = 3.31, *p* < 0.01, with control participants demonstrating a significantly greater increase than patients with IBS immediately after LP

Lembo et al. (2009) [51]	Observational study	1721 patients with IBS and other FGIDs were included only if they met the *Rome II or Rome III diagnostic criteria* for at least 6 months prior to study enrolment and if they had no evidence of an organic, an infectious, or a structural cause of the disease. 10 serum biomarkers were selected from a potential panel of 140 for their ability to differentiate IBS from non-IBS disease in blood samples from patients with IBS and other GI disorders and healthy volunteers	IBS (IBS-C, IBS-D, IBS-M, IBS-U) and non-IBS patients (healthy individuals, IBD, coeliac disease, and functional GI disorders)	876	Blood-based diagnostic test differentiated IBS from non-IBS with 50% sensitivity and 88% specificity, respectively	The overall accuracy of the IBS diagnostic test, defined as the percentage of correct predictions, was 70%. The PPV was 81% and the NPV was 64% at a 50% IBS prevalence

Sidhu et al. (2009) [53]	Observational study	Patients with *Rome II* IBS-D were recruited prospectively	IBS-D	219	*CgA level in serum* appears to be transiently elevated in IBS-D	CgA levels were followed up for a median duration of 7 months. 81% of IBS patients (*n* = 177) had normal CgA levels (0–20 u/L). 12.3% (*n* = 27) had values between 20 and 60 u/L, and 6.8% (*n* = 15) had CgA levels >60 u/L. CgA remained elevated >60 u/L on repeated testing in 3.2% of patients of the whole group

Sidhu et al. (2010) [55]	Observational study	Patients with IBS were diagnosed according to *Rome III criteria.* Duodenal and colonic biopsies were obtained from all IBS patients	IBS-C and IBS-D compared to healthy controls	41	Reduced *density of intestinal CgA cells* as potential histopathological biomarker	Duodenum CgA cell density healthy control versus IBS total (50.5 ± 21 versus 25.6 ± 22, *p* < 0.05). Colon CgA cell density healthy control versus IBS total (33.1 ± 14 versus 21.3 ± 13, *p* < 0.001)

Öhman et al. (2012) [39]	Observational study	No data on diagnostic criteria. Fecal samples for analysis of calprotectin, CgA and CgB, and SgII and SgIII were collected from all study subjects	IBS (IBS-C, IBS-D, and IBS-A) compared to healthy controls	82	Higher levels of CgA and secretogranins II and III in fecal samples	Higher levels of fecal CgA (*p* = 0.009), SgII (*p* < 0.001), and SgIII (*p* < 0.001), but lower levels of CgB (*p* < 0.001) compared with controls

Tibble et al. (2002) [61]	Observational study	602 new referrals to a gastroenterology clinic who had symptoms suggestive of IBS or organic intestinal disease were studied to assess the sensitivity, specificity, and odds ratios (ORs) of fecal calprotectin, small intestinal permeability, *Rome I criteria,* and laboratory markers of inflammation (ESR, CRP, blood count) in distinguishing organic from nonorganic intestinal disease	Patients with IBS symptoms (IBS/IBD)	602 (339/263)	Cutoff *FC level* of 30 mg/kg combined with Rome I criteria can serve as a clear proof of IBS with no need for further examination	At the cutoff value for normality (10 mg/L), fecal calprotectin has a sensitivity of 89% and specificity of 79% for organic disease. Predictive values of FC at >10 mg/L (OR (95% CI) 27.8 (17.6–43.7), PPV 0.76, NPV 0.89)

Waugh et al. (2013) [62]	Systematic review	A broad search strategy was run in several databases. Studies that provided sufficient data for calculation of sensitivity, specificity, and other diagnostic outcomes were Identified. The quality of studies was assessed using Quality Assessment of Diagnostic Accuracy Studies	IBS compared to IBD	28 studies	Cutoff *FC level* from 50 mcg/g showed 93% of sensitivity and 94% of specificity in differentiating IBD from IBS	

Chang et al. (2014) [63]	Observational study	No data on diagnostic criteria for IBS in abstract. 20 healthy control subjects, 26 patients with IBS, and 58 patients with IBD, including 22 with ulcerative colitis (UC) and 36 with Crohn's disease (CD), were recruited. FC was analyzed in stool samples, and CRP and the ESR were assessed in blood samples	IBS/IBD/healthy controls	26	Significantly *higher stool FC levels* in IBS patients than serum CRP levels	In patients with IBD and IBS, significant increases in fecal calprotectin and CRP levels were observed (694.8 ± 685.0 *µ*g/g in IBD versus 85.8 ± 136.1 *µ*g/g in IBS and 0.851 ± 1.200 mg/dL in IBD versus 0.16 ± 0.23 mg/dL in IBS, resp.; *p* < 0.0001)

Tkalcic et al. (2014) [64]	Observational study	Prospectively selected IBS patients diagnosed according to *Rome III criteria.* All patients underwent stool sampling for FC level and fulfilled HRQoL questionnaires (IBS-36)^2^ and (SF-36)^3^	IBS	48	*FC level* correlates with reduced physical component of health related quality of life (HRQoL)	Physical component of HRQoL was predicted by calprotectin (*β* = −0.34; *p* < 0.01), depression (*β* = −0.31; *p* < 0.05), anxiety (*β* = −0.27; *p* < 0.05), and symptom severity (*β* = −0.25; *p* < 0.05)

Langhorst et al. (2009) [65]	Observational study	No data on diagnostic criteria for IBS in abstract. Fecal specimens were collected from a total of 100 participants. Exclusion criteria: current use of probiotics and antibiotics, IBS patients CRP or leukocytes, a history of bacterial overgrowth, or infectious gastrointestinal disease over the last 6 months	IBS and UC and healthy controls	46	Significantly elevated levels of HBD-2 in patients with IBS compared with HCs and similar to those with active UC	HBD-2 levels were highest in active UC (106.9 ± 91.5 ng/g), almost as high in IBS (76.0 ± 67.9 ng/g), and lowest for HCs (29.9 ± 16.1 ng/g). Scheffe post hoc tests revealed significant differences (*p* < 0.001) between the groups of patients (UC and IBS) versus HCs

^1^FBDSI: Functional Bowel Disorder Severity Index, ^2^IBS-36: Irritable Bowel Syndrome Quality of Life Questionnaire, ^3^SF-36: Medical Outcome Study Short Form 36, ^4^TSST: Trier Social Stress Test, ^5^SCL: skin conductance level, and ^6^ACTH: adrenocorticotropic hormone.

## Study Highlights

*What Is Current Knowledge.* (i) Most IBS patients are referred to secondary centers for diagnostic evaluation, despite established IBS diagnostic criteria. (ii) Heterogeneity of symptoms, overlapping symptoms with some serious organic diseases, and unclear understanding of pathophysiology of the disease present problems for researchers and their attempt to establish a universal test to confirm the diagnosis of IBS. (iii) Most of the immunologic and endocrine biomarkers studied are highly unspecific for IBS.

*What Is New*. (i) We review recent studies (2009–2014) that have searched for adequate biomarker for IBS. (ii) There is no strong evidence that any biomarker is adequate for diagnosis confirmation, because most of the reviewed studies were observational. (iii) Examination of the proteome (proteomics) is a promising approach in biomarker discovery.
